# Antimyeloperoxidase antibodies modulate inflammatory responses and activate profibrotic pathways in human monocytes

**DOI:** 10.1016/j.jaut.2023.103060

**Published:** 2023-09

**Authors:** Fernanda Flórez-Barrós, Siobhan Bearder, Polychronis Pavlidis, Michael G. Robson

**Affiliations:** School of Immunology and Microbial Sciences, King's College London, UK

**Keywords:** Vasculitis, ANCA, Autoimmunity, Autoantibody, Inflammation, Monocyte

## Abstract

Antimyeloperoxidase (anti-MPO) and antiproteinase 3 (anti-PR3) antibodies are found in anti-neutrophil cytoplasmic antibody-associated vasculitis (AAV). We investigated the effect of both anti-MPO and anti-PR3 IgG on human monocytes. Peripheral blood monocytes were cultured under a range of conditions that included TLR agonists, anti-MPO IgG and anti-PR3 IgG with appropriate controls. Experiments included whole transcriptome profiling and an assessment of the role of Fc receptors.

When monocytes were stimulated with LPS or R848, anti-MPO but not anti-PR3 IgG, caused a reduction in IL-10 secretion and had a profound effect on cell-surface marker expression. Anti-MPO but not anti-PR3 IgG enhanced monocyte survival in the absence of TLR stimulation. These effects depended on the Fc receptor CD32a. With TLR stimulation, the effect of anti-MPO but not anti-PR3 IgG on the transcriptional response at 6 h was variable, but we identified a core set of transcripts likely to be important. Without TLR stimulation, there was a robust effect of anti-MPO but not anti-PR3 IgG on the transcriptional response at 24 h, and there was a highly significant enrichment of genes encoding extracellular matrix and extracellular matrix-associated proteins. Analysis with nCounter confirmed many of the differentially expressed transcripts and supported a role for CD32a. These data show that anti-MPO, but not anti-PR3 IgG, from patients with AAV has wide-ranging effects on monocytes which depend on CD32a. The activation of a profibrotic transcriptional response by anti-MPO but not anti-PR3 IgG may give insights into the differences in disease phenotype.

## Highlights

• Anti-MPO IgG, but not anti-PR3 IgG, prolonged human monocyte survival and profoundly modified responses to LPS and R848.
• Both effects required the Fc receptor CD32a.
• The transcriptional response of monocytes cultured with anti-MPO IgG was directed towards the expression of extracellular matrix associated proteins.
• These findings may be relevant to inflammation and fibrosis in ANCA vasculitis and suggest transcripts for further study.

## 1 Introduction

Anti-neutrophil cytoplasmic antibody (ANCA) vasculitis is a systemic disease caused by vascular inflammation, with glomerulonephritis and pulmonary haemorrhage being the most serious clinical features [1]. Following the initial description of ANCA [2] it was established that the antigenic targets of the autoantibodies are the proteins myeloperoxidase [3] or proteinase-3 [4] which are found within granules and on the surface of both neutrophils and monocytes. More recently antibodies to lysosomal membrane protein-2 (LAMP-2) have been shown to be highly prevalent in this patient group [5,6]. Passive administration of anti-MPO antibodies causes focal necrotising crescentic glomerulonephritis in mice [7,8], providing evidence to support the pathogenicity of ANCA. ANCA may activate neutrophils to undergo respiratory burst and degranulation as first shown by Falk et al. [9] and confirmed by others (reviewed in Ref. [10]). However, this finding has not always been reproduced. Our own work, with a large selection of ANCA and a number of assays of neutrophil activation, did not show an effect compared to control IgG [11].

Monocytes and macrophages are present at sites of active glomerular disease [12] but the responses of monocytes to ANCA have been less studied than those of neutrophils. The secretion of proinflammatory cytokines such as IL-8, IL-1β and IL-6 from monocytes in response to ANCA has been described [13,14]. Furthermore, the production of oxygen radicals by TNFα-primed monocytes in response to MPO-ANCA or PR3-ANCA has been observed [15]. In a previous publication, we made some preliminary observations on the effect of MPO-ANCA (anti-MPO IgG) on monocytes [16]. We found that MPO-ANCA but not PR3-ANCA (anti-PR3 IgG) inhibited the secretion of IL-10 from LPS-primed peripheral blood monocytes. Furthermore MPO-ANCA significantly increased monocyte survival, whereas the effect of PR3-ANCA on monocyte survival was not assessed.

In view of the uncertainty regarding the effects of ANCA on neutrophils [11] and the central role that monocytes have in both inflammation and fibrosis, we have extended this preliminary work. The objectives of the current study were to (a) examine cell-surface marker expression on human monocytes in response to anti-MPO or anti-PR3 IgG with TLR stimulation (b) extend our analysis to include other TLR agonists in addition to LPS, (c) explore whether the effect of anti-MPO IgG on monocyte survival was seen with anti-PR3 IgG, (d) explore the role of specific Fc receptors in any observes effects, and (e) perform whole transcriptome profiling to examine genes and pathways mediating the effects of anti-MPO IgG on human monocytes. None of these objectives were included in our previous report.

## 2 Methods

### 2.1 Study approval

Samples from patients and healthy donors were taken following informed consent with ethical approval (NRES committee London—London Bridge 09/H084/72).

### 2.2 Purification of IgG from patients and controls

Blood or plasma exchange fluid was obtained from patients with ANCA vasculitis and circulating MPO- or PR3-ANCA. Blood was also obtained from healthy controls. Plasma or serum was stored at −80 °C. Fibrinogen was precipitated by adding 18 g/100 ml of sodium chloride and IgG was purified with protein G chromatography (GE Healthcare). The endotoxin concentration in the final IgG preparations was measured by Lonza using a LAL kinetic chromogenic assay. For all polyclonal IgGs used, the endotoxin was less than 0.05 eU/250 μg IgG. The concentration of IgG used for all assays was 250 μg/ml. Anti-MPO and anti-PR3 levels in purified IgG preparations were measured in the clinical immunology laboratory at Kings’ College Hospital (Viapath), using the FIDIS™ Luminex based assay. Before all assays, IgG preparations were centrifuged at 16,000 g for 30 min at 4 °C to remove aggregates.

### 2.3 Monocyte isolation and culture

Peripheral blood mononuclear cells from healthy controls were isolated from blood anticoagulated with EDTA using Ficoll-Paque (GE Healthcare) density gradient centrifugation. For experiments including TLR agonists, monocytes were then isolated by depletion of CD16^+^ cells followed by positive section for CD14^+^ cells with magnetic microbeads (Miltenyi Biotec). For experiments performed not including TLR agonists, cells were positively selected for CD14^+^, but not CD16 depleted. This was because, in preliminary experiments without TLR agonists, the effects of anti-MPO IgG were not as clearly seen with CD16 depleted monocytes. Monocytes were resuspended in RPMI and 10% AB serum (experiments not including TLR agonists) or 10% FCS (experiments including TLR agonists). Monocytes were plated as 500 μl at 1 × 10^6^ cells/ml in 48 well plates or at 170 μl at 1 × 10^6^ cells/ml in 96 well plates as indicated. Where indicated, LPS (25 ng/ml, *Escherichia coli* serotype R515 (Re), TLRgrade®, Enzo Life Sciences, UK) or R848 (5 μg/ml, Invivogen) was added. 250 μg/ml of control, anti-PR3 or anti-MPO IgG was added and plates incubated at 37 °C for the time indicated. Where data for cell numbers are given, cells were detached using ice-cold PBS-EDTA and counted using Trypan blue (to exclude dead cells) and a haemocytometer. Where indicated Fc receptor blocking antibodies were added at 5 μg/ml. This concentration was based on preliminary experiments. These antibodies were anti-CD32a (clone IV.3, Stem Cell), anti-CD32b (clone Ch2B6N297Q, MacroGenics) and anti-CD64 (clone 10.1, BD Biosciences). Each monocyte donor is assigned a letter which indicates the same monocyte donor in different experiments.

### 2.4 Flow cytometry

After 18 h, supernatants were collected and cells detached using ice-cold PBS-EDTA, and stained using the following conjugated antibodies for surface markers: CD14 (clone M5E2, Biolegend), CD16 (clone NKP15, BD Biosciences), CD25 (clone M-A251, BD Biosciences), CD11b (clone ICRF44, Biolegend), CD80 (clone L307.4, BD Biosciences), CD69 (clone FN50, Biolegend). Dead cells were excluded with with Fixable Viability Dye BV421 (BD Biosciences). Samples were run on a BD Fortessa flow cytometer and data analysed using FlowJo software (BD Biosciences).

### 2.5 Cytokine ELISAs

Supernatants were used to measure IL-6, IL-10, IL-1β, IL-12, MIP-1α, TNF-α, MPO and PR3 by ELISA (Biotechne DuoSet) following the manufacturer's instructions.

### 2.6 RNA-seq transcriptional analysis

Peripheral blood monocytes were isolated from healthy donors and cultured as described above in 48 well plates. Purified IgG or TLR agonists were included in the culture as indicated. At 6 h (experiments with TLR agonists) or 24 h (experiments without TLR agonists) RNA was purified using Monarch total RNA miniprep kit (New England BioLabs Inc.) according to manufacturer's instructions.

Library preparation and sequencing was performed by Novogene Bioinformatics Technology (Beijing, PR China). Sequencing libraries were generated using NEBNext® UltraTM RNA Library Prep Kit for Illumina® (NEB, USA) following manufacturer's recommendations and index codes were added to attribute sequences to each sample. Briefly, mRNA was purified from total RNA using poly-T oligo-attached magnetic beads. Fragmentation was carried out using divalent cations under elevated temperature in NEBNext First Strand Synthesis Reaction Buffer (5X). First strand cDNA was synthesized using random hexamer primer and M-MuLV Reverse Transcriptase (RNase H-). Second strand cDNA synthesis was subsequently performed using DNA Polymerase I and RNase H. Remaining overhangs were converted into blunt ends via exonuclease/polymerase activities. After adenylation of 3′ ends of DNA fragments, NEBNext Adaptor with hairpin loop structure were ligated to prepare for hybridization. In order to select cDNA fragments of preferentially 150–200 bp in length, the library fragments were purified with AMPure XP system (Beckman Coulter, Beverly, USA). Then 3 μl USER Enzyme (NEB, USA) was used with size-selected, adaptor-ligated cDNA at 37 °C for 15 min followed by 5 min at 95 °C before PCR. Then PCR was performed with Phusion High-Fidelity DNA polymerase, Universal PCR primers and Index (X) Primer. Next, PCR products were purified (AMPure XP system) and library quality was assessed on the Agilent Bioanalyzer 2100 system. The clustering of the index-coded samples was performed on a cBot Cluster Generation System using PE Cluster Kit cBot-HS (Illumina) according to the manufacturer's instructions. After cluster generation, the library preparations were sequenced on a Novaseq 600 machine (Illumina platform) and paired-end reads were generated. The read depth was 30 million reads per sample.

Raw data (raw reads) of FASTQ format were firstly processed through in-house scripts. In this step, clean data (clean reads) were obtained by removing reads containing adapter and poly-N sequences and reads with low quality from raw data. At the same time, Q20, Q30 and GC content of the clean data were calculated. All the downstream analyses were based on the clean data with high quality. Reference genome and gene model annotation files were downloaded from genome website browser (NCBI/UCSC/Ensembl) directly. Paired-end clean reads were aligned to the reference genome using the Spliced Transcripts Alignment to a Reference (STAR) software, which is based on a previously undescribed RNA-seq alignment algorithm that uses sequential maximum mappable seed search in uncompressed suffix arrays followed by seed clustering and stitching procedure. HTSeq was used to count the read numbers mapped of each gene. RPKM of each gene was then calculated based on the length of the gene and reads count mapped to this gene. RPKM, Reads Per Kilobase of exon model per Million mapped reads, considers the effect of sequencing depth and gene length for the reads count at the same time. The data discussed in this publication have been deposited in NCBI's Gene Expression Omnibus and are accessible through GEO Series accession number GSE220695 (https://www.ncbi.nlm.nih.gov/geo/query/acc.cgi?&acc=GSE220695).

Volcano plots and heat maps were generated using Prism version 9 (Graphpad). Venn diagrams were generated using this URL: https://bioinformatics.psb.ugent.be/webtools/Venn/. Transcript lists were analysed with Metascape [17] (open source), using the express analysis option. Pathway and process enrichment analysis used the following ontology sources: KEGG Pathway, GO Biological Processes, Reactome Gene Sets, Canonical Pathways, CORUM, WikiPathways and PANTHER Pathway. All genes in the genome were used as the enrichment background. The analysis date was 27 October 2022.

### 2.7 Nanostring transcriptional analysis

Peripheral blood monocytes were isolated from healthy donors and cultured as described above in 48 well plates. Purified IgG, TLR agonists or Fc receptor blocking antibodies were included in the culture as indicated. At 6 h (experiments with TLR agonists) or 24 h (experiments without TLR agonists) RNA was purified using Monarch total RNA miniprep kit (New England BioLabs Inc.) according to manufacturer's instructions. Using the nCounter system (Nanostring technologies), a custom codeset was obtained and samples were run at the Nanostring facility, University College London. Nanostring data was processed and analysed as recommended by the company. The geometric mean of the synthetic positive controls was calculated for each lane. A lane-specific normalisation factor was then calculated by dividing the geometric mean for a given lane by the arithmetic mean of all of the geometric means. A similar calculation was then used to normalise for 3 housekeeping genes.

### 2.8 Statistics

Prism version 9 (Graphpad) was used unless other software is specified. Where two groups were compared a student's t-test was used and for more than two groups a one-way ANOVA was used as indicated in the figure legends. Cytokine and flow cytometry data (but not cell count data) were logarithmically transformed prior to analysis. Differential expression analysis for the RNA-seq data was performed by Novogene using DESeq2 (open source software), based on a model using the negative binomial distribution with p values adjusted for false-discovery using the method of Benjamini and Hochberg. In Metascape [17], p-values were calculated based on the cumulative hypergeometric distribution and q-values are calculated using the Benjamini-Hochberg procedure to account for multiple testings. Analysis was performed using differentially expressed gene lists for each monocyte donor and separately and for a list produced by merging lists from the three monocyte donors. For analysis of the Nanostring data, Log2 data were analysed in Prism version 9 (Graphpad) using multiple unpaired t tests with Benjamini, Krieger, and Yekutieli's correction for false discovery.

## 3 Results

### 3.1 Antimyeloperoxidase antibodies modulate the cytokine response to TLR agonists

**Fig. 1:**
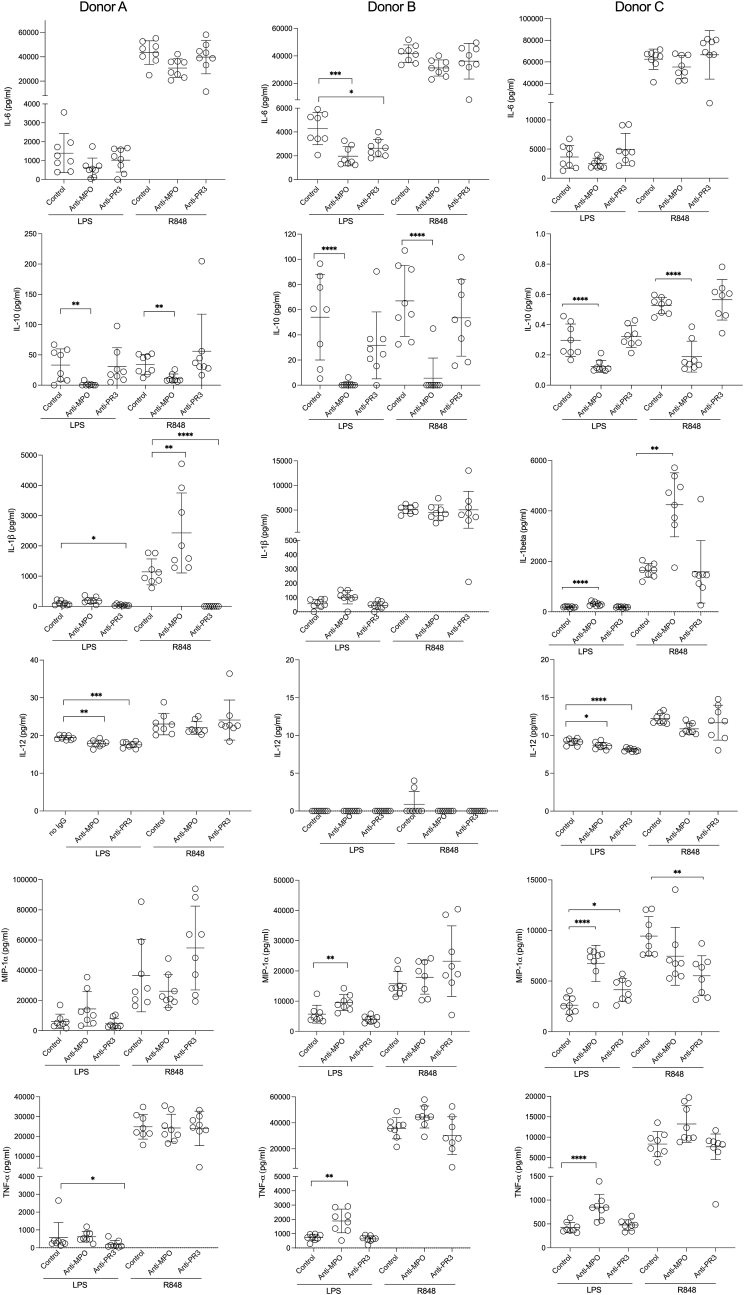
Cytokine secretion from human peripheral blood monocytes stimulated with TLR agonist and anti-MPO, anti-PR3 or control IgG. Monocytes were incubated in 48 wells plates for with LPS or R848, in addition to the indicated IgG (n = 8 per group) for 18 h. Cytokines were measured in supernatants at 18 h. Symbols represent different IgG preparations and not technical replicates. Experiments were performed with monocytes from 3 separate healthy donors. LPS and R848 treated groups were analysed separately using a one-way ANOVA with Dunnett's post-test. Anti-MPO and anti-PR3 groups were compared with the control group. Error bars are mean (SD). *p < 0.05, **p < 0.01, ***p < 0.001, ****p < 0.0001.

In the current report, IgG preparations were from different patients and controls to those who provided samples in our previous publication [16]. Patient characteristics, and the anti-MPO and anti-PR3 levels in purified IgG preparations are shown in Table S1. As seen previously [16], there was no consistent effect of anti-PR3 IgG but a marked reduction in IL-10 with anti-MPO IgG (Fig. 1). We extended our previous observations by stimulating monocytes with the TLR7/8 agonist R848 in addition to LPS. We found a consistent reduction in IL-10 in the presence of anti-MPO IgG with either LPS or R848 in all 3 healthy monocyte donors, after 18 h of incubation (Fig. 1). No consistent differences between groups were seen for other cytokines. Furthermore, there was no consistent effect for anti-PR3 IgG. These data show that the previously demonstrated modulation of the response to TLR stimulation by anti-MPO IgG is reproducible in monocytes after CD16^+^ cell depletion and with a new panel of IgG preparations. Furthermore, it is not specific to TLR4 stimulation by LPS, because it was also seen with TLR7/8 stimulation by R848.

### 3.2 Antimyeloperoxidase antibodies modulate expression of cell-surface markers in response to TLR agonists

**Fig. 2:**
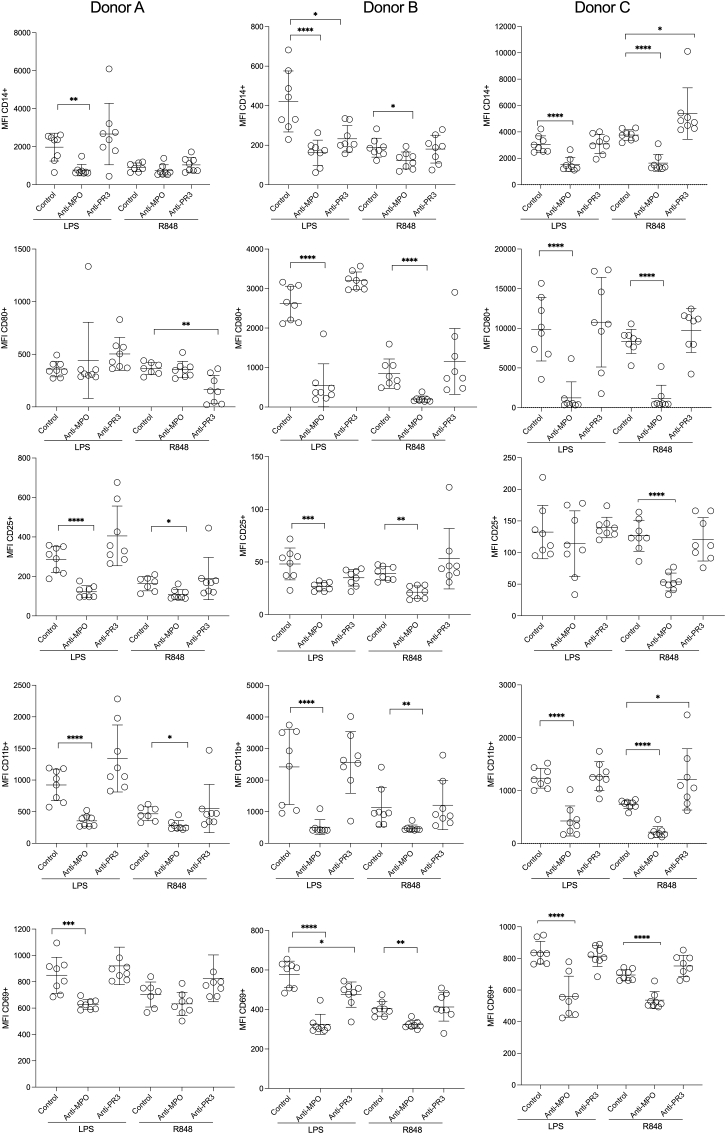
Flow cytometric analysis of human peripheral blood monocytes stimulated with TLR agonist and anti-MPO, anti-PR3 or control IgG. Monocytes were incubated in 48 well plates with LPS or R848, in addition to the indicated IgG (n = 8 per group, except donor A /R848/control where (for technical reasons) n = 7). The gating strategy is shown in Supplementary Fig. S2. Symbols represent different IgG preparations and not technical replicates. Experiments were performed with monocytes from 3 separate healthy donors. MFI: Median fluorescence intensity. LPS and R848 treated groups were analysed separately using a one-way ANOVA with Dunnett's post-test. Anti-MPO and anti-PR3 groups were compared with the control group. Error bars are mean (SD). *p < 0.05, **p < 0.01, ***p < 0.001, ****p < 0.0001.

In order to provide a more comprehensive assessment of the monocyte response to TLR agonists and ANCA, we investigated cell-surface marker expression. The gating strategy is shown in Supplementary Fig. S2. All 3 donors had reductions in CD11b with anti-MPO IgG and both LPS and R848 (Fig. 2). All 3 donors had reductions in both CD69 and CD14 with anti-MPO IgG and LPS and 2 donors also had a reduction in CD69 and CD14 with anti-MPO IgG and R848. MPO-ANCA caused reductions in CD80 in 2 of 3 donors with both LPS and R848 stimulation. Anti-MPO IgG caused a reduction in CD25 in 2 donors with LPS stimulation, and in all 3 donors with R848 stimulation. There were no consistent effects seen due to anti-PR3 IgG with either LPS or R848. Overall, these data show a profound effect on classical monocyte phenotype due to anti-MPO IgG but not anti-PR3 IgG, in the context of stimulation with either LPS or R848.

### 3.3 Antimyeloperoxidase antibodies prolong monocyte survival

**Fig. 3:**
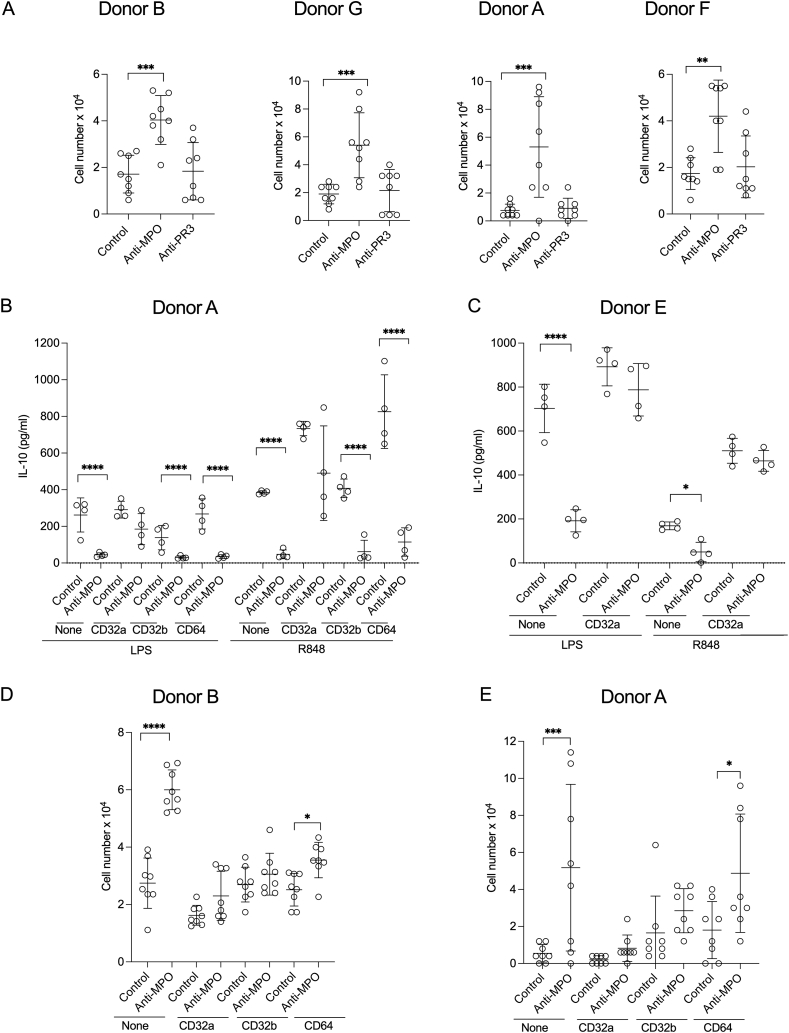
A. The survival of human peripheral blood monocytes stimulated with anti-MPO, anti-PR3 or control IgG. B-E. The effect of Fc receptor blockade on the responses of human peripheral blood monocytes to anti-MPO, anti-PR3 or control IgG. For A and D-E, Monocytes were cultured with the indicated IgG (n = 8 per group) in 96 well plates with or without the blocking antibody indicated for 6 days. The starting cell number was 1.7 × 10^5^ cells and the total cell number remaining at day 6 was counted. For B–C, monocytes were incubated for 18 h in 96 well plates with LPS or R848, with or without the blocking antibody indicated, in addition to IgG from patients or controls for 18 h (n = 4 per group). IL-10 was measured in supernatants at 18 h. Symbols represent different IgG preparations and not technical replicates. For A, a one-way ANOVA with Dunnett's post-test was used and anti-MPO and anti-PR3 groups were compared with the control group. For B–C, LPS and R848 treated groups were analysed separately. For B-E, a one-way ANOVA with Sìdák's post-test was used, with pairs of anti-MPO and control groups, (treated with none, anti-CD32a, anti-CD32b or anti-CD64) selected for comparison. Error bars are mean (SD). *p < 0.05, **p < 0.01, ***p < 0.001, ****p < 0.0001.

There was a clear increase in the number of monocytes remaining after 6 days in culture with anti-MPO IgG compared to control (Fig. 3A) as seen previously [16]. The effect was reproduced in four different monocyte donors and was not seen with anti-PR3 IgG. After 6 days, the concentration of MPO protein in the supernatants was approximately 5 times higher than PR3 (Supplementary Fig. S3). Anti-MPO IgG and anti-PR3 IgG would have interfered with the ELISA and so data for these groups are not shown. These results confirm the effect of anti-MPO IgG on monocyte survival and further show there is no effect of anti-PR3 IgG. The data for anti-PR3 IgG are novel as these experiments were not included in our previous study.

### 3.4 The effects of antimyeloperoxidase antibodies on monocytes require CD32a

In view of the importance of Fc receptors for IgG immune-complex mediated monocyte functions, we assessed their role in the effects of anti-MPO IgG. There are three classes of Fcγ receptor for IgG on circulating monocytes. These are FcγI (CD64), FcγII (CD32) and FcγIII (CD16) [18]. FcγIIa (CD32a) is a stimulatory receptor with FcγIIb (CD32b) being inhibitory. CD16^+^ depleted monocytes were used for the experiments with TLR agonists and so we did not consider CD16 further. Blockade of CD32a inhibited the reduction in IL-10 secretion due to anti-MPO IgG, whereas blockade of CD32b or CD64 had no effect (Fig. 3B). This effect of CD32a blockade was seen with both LPS and R848 and was confirmed in a second monocyte donor (Fig. 3C). Fc receptors were also required for the effect of anti-MPO IgG on monocyte survival. The increase in survival was inhibited with blockade of CD32a (Fig. 3D–E). In addition, the effect of anti-MPO IgG on monocyte survival was reduced with blockade of CD32b but not CD64. The results were shown in two healthy monocyte donors (Fig. 3D–E). Overall, CD32a was required for all of the observed effects of anti-MPO IgG.

### 3.5 Transcriptional analysis of the response to TLR agonists and antimyeloperoxidase antibodies

**Fig. 4:**
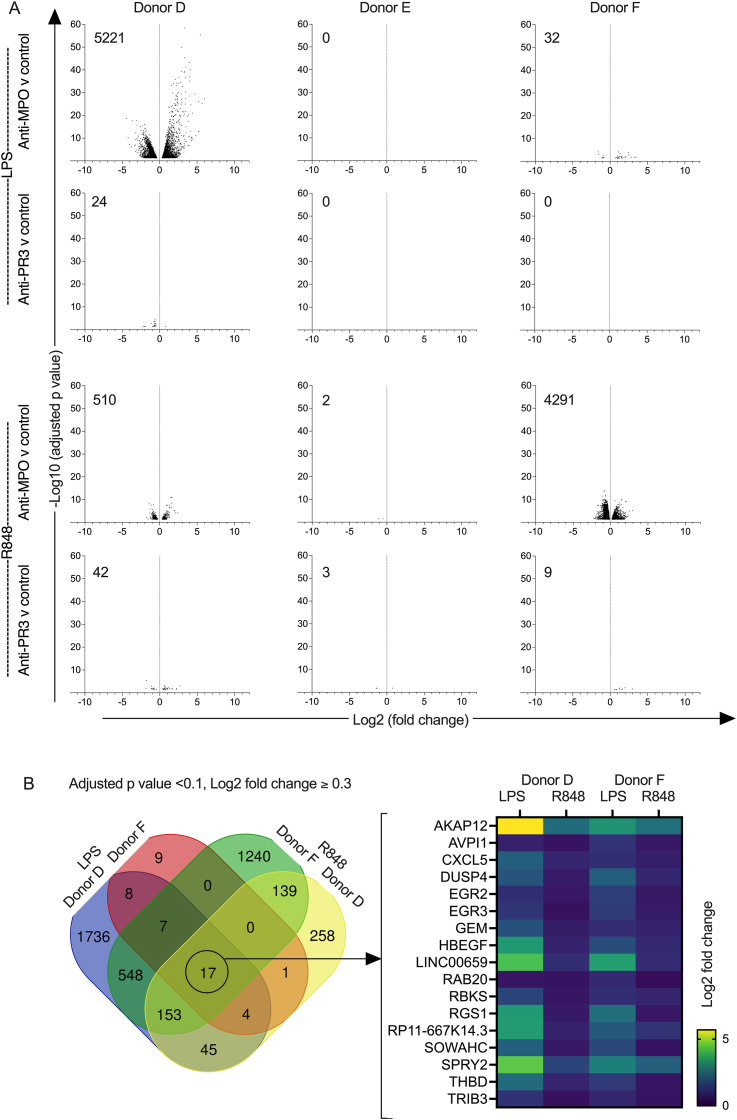
RNA-seq analysis of human peripheral blood monocytes stimulated for 6 h with TLR agonist and anti-MPO, anti-PR3 or control IgG. A. Volcano plots of differentially expressed transcripts when samples stimulated with anti-MPO or anti-PR3 IgG were each compared with samples stimulated with control IgG (n = 8 per group except in 3 of the 18 groups n = 7 for technical reasons. These were control/donor D/LPS, anti-MPO/donor F/R848 and anti-PR3/donor F/LPS). Experiments were performed in the presence of stimulation with R848 or LPS, and with three different healthy monocyte donors. Transcripts with an adjusted p value of <0.05 are shown with the total number of transcripts shown indicated on each plot. B. Venn diagram showing the transcripts differentially upregulated with anti-MPO IgG (versus control IgG), with an adjusted p value of less than 0.1 and a log2 fold change of at least 0.3. There were 17 transcripts that were identified as differentially expressed in both donors D and F in the presence of both LPS and R848. These transcripts are listed and shown in a heat map of fold change.

We performed whole transcriptome profiling under the experimental conditions used for the experiments in Fig. 1, Fig. 2. RNA was isolated after 6 h in experiments using monocytes from three different donors. Volcano plots are shown in Fig. 4A, with anti-MPO IgG and anti-PR3 IgG groups each compared to the control IgG group. There was a marked variation in the number of differentially expressed transcripts seen with each donor at 6 h for both LPS and R848 treated monocytes (Fig. 4A). As the effects of anti-MPO IgG on IL-10 secretion and cell surface marker expression at 18 h were consistent (Fig. 1, Fig. 2), it seemed likely that we had captured the early transcriptional response in donor D for LPS, and in donor F for R848. Given the low numbers of differentially expressed genes, we used relaxed thresholds of an adjusted p value of less than 0.1 and a log2 fold change of 0.3. Lists of trancripts upregulated with anti-MPO IgG compared to control IgG using these criteria are shown in supplementary data (transcript list 1). A Venn diagram constructed from these lists is shown in Fig. 4B. There were 17 transcripts that were differentially upregulated with anti-MPO IgG compared to control IgG in monocyte donors D and F with both LPS and R848.

### 3.6 Transcriptional analysis of the response to antimyeloperoxidase antibodies in the absence of TLR stimulation

**Fig. 5:**
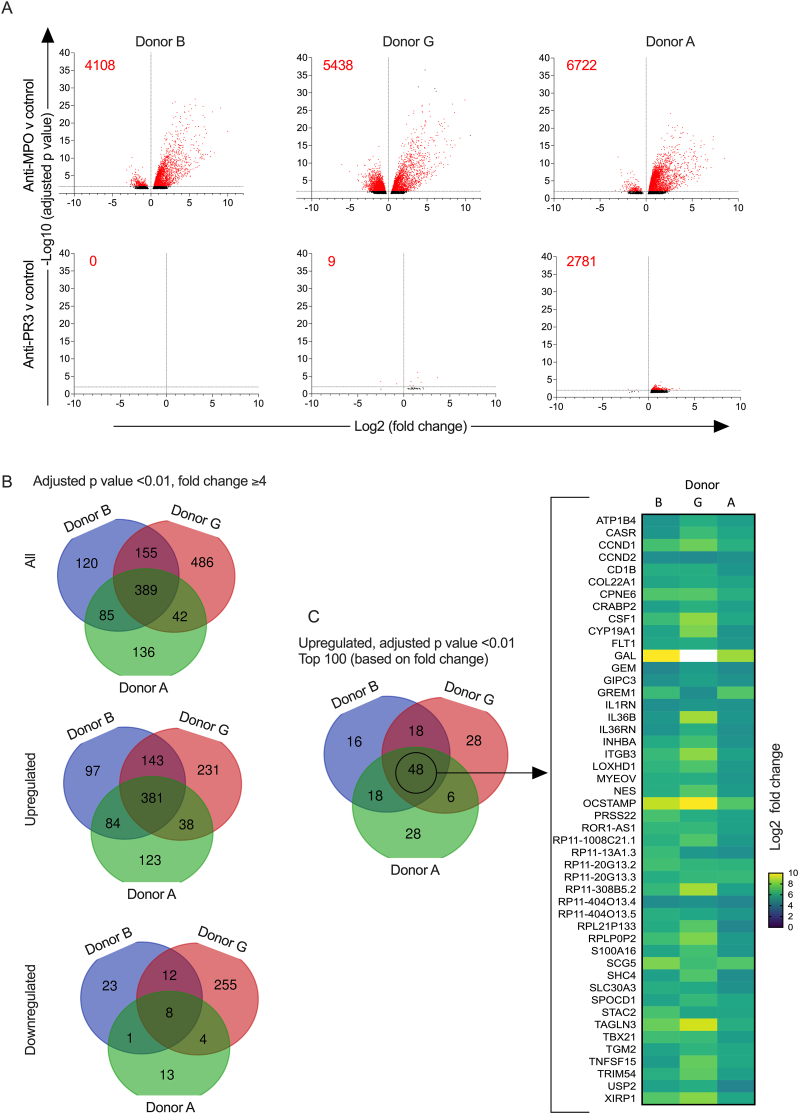
RNA-seq analysis of human peripheral blood monocytes (from three healthy monocyte donors) cultured with anti-MPO, anti-PR3 or control IgG for 24 h. A. Volcano plots of differentially expressed transcripts when samples stimulated with anti-MPO IgG or anti-PR3 IgG were each compared with samples stimulated with control IgG (n = 8 per group). Transcripts with an adjusted p value of <0.05 are shown and those with an adjusted p value of <0.01 are red. The number in red for each plot indicates the total number of transcripts with an adjusted p value of <0.01. B. Venn diagrams showing the differentially expressed transcripts (all, upregulated or downregulated with anti-MPO IgG versus control IgG) in the three monocyte donors with an adjusted p value < 0.01 and a fold change of at least 4. C. Venn diagram showing the top 100 transcripts (based on fold change of those with an adjusted p value < 0.01) upregulated with anti-MPO IgG versus control IgG for each donor. There were 48 transcripts that were identified as upregulated in experiments with all 3 monocyte donors and these are listed and shown in a heat map of fold change.

We performed whole transcriptome profiling of the effect of anti-MPO IgG on monocyte phenotype under the experimental conditions used for the experiments in Fig. 3A. RNA was isolated after 24 h in experiments using monocytes from three different donors. Volcano plots are shown in Fig. 5A, with anti-MPO IgG and anti-PR3 IgG groups each compared to the control IgG group. Given the large number of differentially expressed transcripts with anti-MPO IgG in all donors (Fig. 5A), we set stringent thresholds for transcript list analysis, with an adjusted p value of less than 0.01 and a fold change of at least 4 for both over- and under-expressed transcripts. Lists of transcripts upregulated or downregulated, with anti-MPO IgG compared to control IgG, using these criteria are shown in supplementary data (transcript list 2). Venn diagrams, constructed from these lists are shown in Fig. 5B, with separate diagrams for all transcripts, upregulated transcripts and downregulated transcripts. Of note, none of the differentially expressed transcripts for donor A with anti-PR3 IgG met these criteria. We constructed another Venn diagram from the top 100 upregulated transcripts for each donor (based on fold change) as shown in Fig. 5C. The overlap across monocyte donors persisted and we identified 48 transcripts that were upregulated in all three monocyte donors. These are shown in a heat map of fold change.

**Fig. 6:**
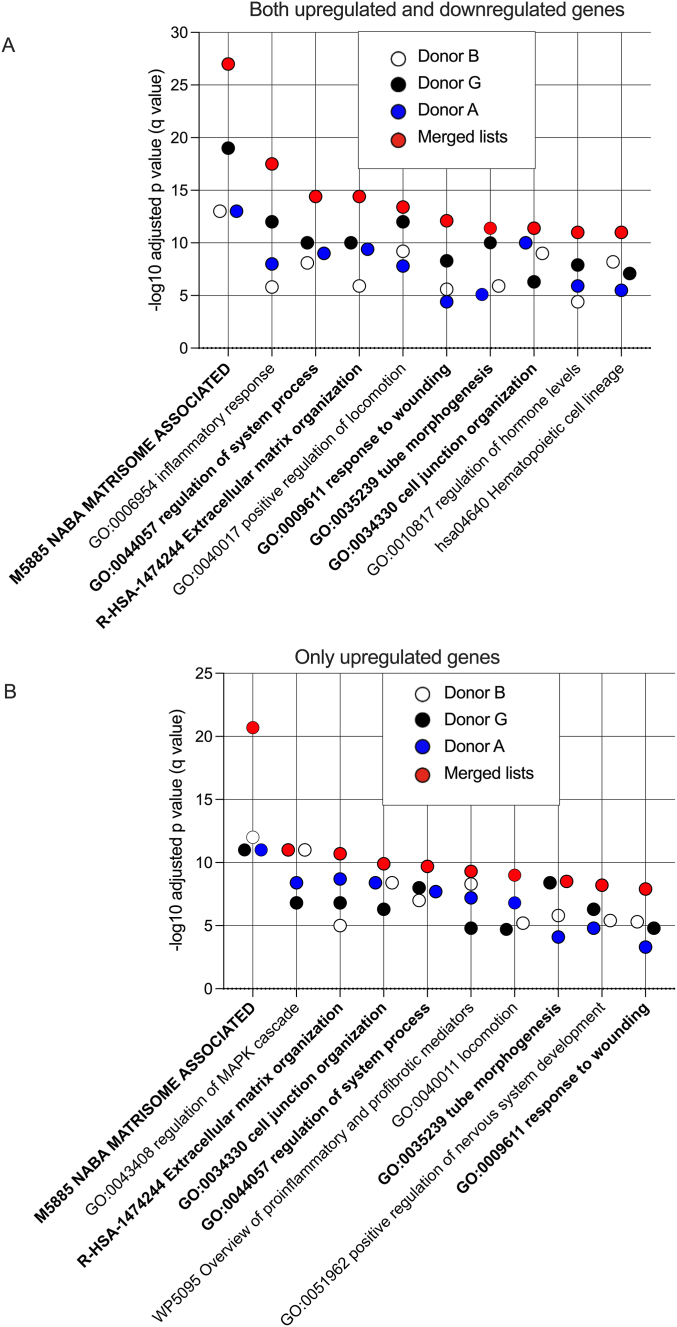
Pathway and process enrichment analysis using Metascape for the experiment shown in Fig. 5. Transcript lists for the Metascape input were obtained using an adjusted p value of less than 0.01 and a fold change of at least 4 as shown in the Venn diagrams in Fig. 5B. A. Results using all transcripts differentially expressed (upregulated or downregulated) with anti-MPO IgG versus control IgG. B. Results using only transcripts upregulated with anti-MPO IgG versus control IgG. Bold type indicates those present in A and B. P-values are calculated based on the cumulative hypergeometric distribution and q-values are calculated using the Benjamini-Hochberg procedure.

Pathway and process enrichment analysis was performed with Metascape for transcript lists used to construct the Venn diagrams in Fig. 5B (adjusted p value of less than 0.01 and fold change of 4 or more). For all differentially expressed transcripts (both up and down), Metascape identified 619, 882 and 561 unique genes for donors B, G and A respectively. The top 10 identified pathways are shown in Fig. 6A. For differentially upregulated transcripts, Metascape identified 581, 654 and 537 unique genes for donors B, G and A respectively. The top 10 identified pathways are shown in Fig. 6B. The results for both analyses were similar with pathways identified in both analyses shown in bold. NABA matrisome was the most significantly enriched pathway by several orders of magnitude in both cases.

### 3.7 Nanostring nCounter analysis in the presence and absence of anti-CD32a

In order to validate the most strongly upregulated transcripts from the RNA-seq analysis we performed Nanostring nCounter experiments. We performed this in the presence or absence of anti-CD32a in order to assess if anti-CD32a inhibited the identified transcriptional pathways, in addition to changes in IL-10 or cell survival that had been shown to be modified by anti-CD32a in Fig. 3B–E. Monocytes were stimulated with R848 and anti-MPO or control IgG for 6 h. We measured expression of the 17 transcripts identified as the core set from the RNA-seq experiment (Fig. 4B). We considered differences to be significant when both the p value and the adjusted p value were less than 0.05. There were significant differences in 13 of 17 transcripts for donor A as shown in Fig. S4. Furthermore, in the presence of anti-CD32a, these p values were at least two-fold higher (indicating a less significant difference) for 10 of these 13 genes (shown in bold, Fig. S4A). In contrast, there were no significant differences seen for donor E. The variability in upregulated genes between donors is consistent with the variability in the RNA-seq data (Fig. 4A). Despite this, there was a consistent difference in IL-10 expression that was reversed with anti-CD32a (Fig. 3B–C). These data show that at least 10 transcripts are likely to be key in the modulation of the response of human monocytes to TLR agonists by anti-MPO IgG.

Monocytes were cultured for 24 h with anti-MPO IgG or anti-PR3 IgG, in the presence or absence of anti-CD32a. We measured expression of the top 45 transcripts that were identified as differentially expressed in donors B, G and A (Fig. 5C). For 3 of the 48 transcripts shown in Fig. 5C, Nanostring probes were not available. These were RP11-404O13.4, RP11-308B5.2 and RPL2P133. There were significant differences (both p and adjusted p < 0.05) in 23 of 45 transcripts for donor A, and in 24 of 45 for donor B (Fig. S5). 20 transcripts were significantly upregulated for both donor A and donor B and are shown in bold (Fig. S5A). Treatment with anti-CD32a removed or decreased the significance of the differences in expression (p value at least two-fold higher) for all of the upregulated transcripts for donor B and in all except 6 for donor A (denoted by an asterix in Fig. S5A). These data show that at least 20 transcripts are likely to be key in the activation of profibrotic pathways in human monocytes by anti-MPO IgG.

## 4 Discussion

In the current work we have extended our previous findings [16]. We used CD16 depleted classical monocytes and showed that the modulation of responses to TLR stimulation are found in this specific monocyte subset. In addition, a flow cytometric analysis showed consistent effects on cell-surface marker expression indicating a profound change in monocyte phenotype which is not limited to a reduction in IL-10 secretion. Furthermore, we used a second TLR agonist R848 which activated TLR7 and TLR8 and showed similar effects. Therefore the effects are not limited to TLR4 stimulation. Oxidised phospholipids act as TLR4 antagonists [19] and in our previous publication [16] we suggested the generation of oxidised phospholipids at the monocyte surface may be responsible for the effects seen. Oxidised phospholipid inhibition of TLR signaling is restricted to TLR2 and TLR4 [20]. Therefore the current work, showing effects for TLR7 and/or TLR8, contradicts this previous theory and so we did not pursue it further. The effect on monocyte survival was not seen with anti-PR3 IgG but was specific to anti-MPO IgG and this is another new finding. Blockade of FcγIIa (CD32a) inhibited the effects of anti-MPO IgG both with and without TLR stimulation. This suggests that the effects of anti-MPO IgG are mediated by immune complexes binding specifically to CD32a. CD32a expression on circulating monocytes is reduced in patients with SLE compared to controls [21], suggesting a potential role in disease. However, it is not known if this occurs in patients with ANCA vasculitis.

Despite the robust modulation of monocyte phenotype at 18 h, modulation of the transcriptional response at 6 h was variable for both LPS and R848. The most likely explanation is that this reflects the early time point chosen. Only 46 transcripts were differentially upregulated in donor F with LPS, using an adjusted p value less than 0.1 and log2 fold change of at least 0.3 (Fig. 4B). However, 36 of these were also differentially upregulated in donor D who had 2518 transcripts differentially upregulated in total. Similarly, 309 out of 617 transcripts that were differentially upregulated in donor D with R848 were included in the 2104 transcripts that were differentially upregulated in a donor F. This remarkable overlap is shown in Fig. 4B. The fact that with, either LPS or R848, a third donor showed no differentially upregulated transcripts further supports the suggestion that the variability seen reflected the choice of an early timepoint. We did not examine the transcriptional response at 24 h and assessment at a single timepoint is a limitation of our data. We would predict a more uniform response in different donors at this 24 h, given the robust phenotype seen on flow cytometry (Fig. 2). This variability at 6 h may have had a positive aspect and allowed us to identify transcripts important in the early response to anti-MPO IgG and TLR stimulation. Furthermore, the finding that 17 transcripts were differentially upregulated in both donors with either LPS or R848 led us to consider these as particularly relevant. The Nanostring analysis confirmed this, with differential upregulation due to anti-MPO IgG with R848 stimulation, for 13 transcripts (Fig. S4) and inhibiton by blockade of anti-CD32a in 10 of these. These therefore represent a set of candidate transcripts for the upstream effects of MPO-ANCA on monocytes in the setting of TLR stimulation.

We found a reproducible transcriptional response at 24 h when monocytes were cultured without LPS or R848 but with Anti-MPO IgG (Fig. 5A–C). We selected 24 h as we were exploring a phenotype seen at 6 days. However, the selection of a single timepoint is a limitation of our data. Pathway and process enrichment analysis using Metascape showed enrichment for the NABA matrisome associated gene set. This was the most significant effect by several orders of magnitude and was maintained when the analysis was performed for all genes or for just upregulated genes (Fig. 5A–B). Enrichment for two other gene sets that was conserved in both of these analyses included extracellular matrix organization and response to wounding. Other gene lists that were enriched in both analyses were cell junction organization, regulation of system processes and tube morphogenesis. These pathways and processes are unlikely to be relevant to the effects of anti-MPO IgG on monocytes. However, the dominant effect on the NABA matrisome associated gene set indicates that the phenotype of monocytes cultured with anti-MPO IgG is clearly directed towards the expression of extracellular matrix and extracellular matrix-associated proteins.

Fibrosis is a key mechanism in ANCA vasculitis leading to irreversible injury in kidneys and lungs. A key aim of therapy is resolution of inflammation while minimising scarring. The presence of glomerular sclerosis has been shown to have a major effect on the renal outcome [22]. Lung fibrosis is in particular associated with MPO-ANCA rather than PR3-ANCA [23,24]. Our demonstration that anti-MPO IgG but not anti-PR3 IgG activates fibrosis pathways in monocytes may give some insight into this difference in disease phenotype. After considering the top 100 upregulated transcripts in all three monocyte donors (Fig. 6C), we selected 45 transcripts for a nanostring analysis (Fig. S5) and approximately 50% of these were upregulated with anti-MPO IgG in one or both monocyte donors. The list was narrowed down to 20 transcripts that were upregulated in both donors with a dependance on CD32a in one or both (shown in bold in Fig. S5A). These therefore represent a set of candidate transcripts for the upstream effects of Anti-MPO IgG on monocyte survival in the absence of TLR stimulation.

GTP binding protein overexpressed in skeletal muscle (GEM) showed CD32a dependent upergulation in the nanostring analysis both in the presence of TLR stimulation (Fig. S4) and the absence (Fig. S5). GEM is a GTP-binding protein associated with the inner plasma membrane and this location is in keeping with a potential role in transducing Fc receptor dependant signals due to MPO-ANCA [25]. A recent transcriptional analysis compared peripheral blood samples from patients with chronic obstructive pulmonary disease (COPD) with normal controls. The results suggested that GEM is related to COPD and macrophage polarization [26]. This adds to the suggestion that MPO-ANCA may have a specific role in the pathogenesis of lung disease.

We have found and described wide-ranging effects of IgG from patients with MPO-ANCA but not PR3-ANCA. Although (Table S1) there was a trend towards higher anti-MPO levels (for MPO-ANCA patients) than anti-PR3 levels (for PR3-ANCA patients) in the purified IgG preparations used, there was no signficant difference and this seems unlikely to be the explanation. We detected higher concentrations of MPO than PR3 protein in monocyte culture supernatants (Supplemental Fig. S3) although this converts to similar molar concentrations. As MPO is approximately 5 times the molecular weight of PR3, it would form larger immune complexes. Therefore it is possible that the ability of anti-MPO IgG to form larger immune complexes than anti-PR3 IgG and hence bind to Fc receptors on monocytes is a factor in the effects that are seen with anti-MPO IgG but not anti-PR3 IgG.

We have discussed above the possible relevance of our findings for lung and renal disease. A second difference in clinical phenotype between patients with MPO-ANCA and PR3-ANCA is the presence of granulomatous inflammation in the latter which is referred to as granulomatosis with polyangiitis [27]. Therefore it is possible that MPO-ANCA someonehow protects from chronic inflammation and granuloma formation with an associated increase in profibrotic pathways. Future work will be required to establish clinical relevance of our in vitro findings. We acknowledge that this paper leaves many questions unanswered. We have not identified the precise molecular mechanism by which MPO-ANCA exerts effects on human monocytes. However we have produced a small list of candidate transcripts for each effect and these are a clear basis for further study.

## 5 Conclusions

Anti-MPO IgG, but not anti-PR3 IgG, caused a profound change in the phenotype of monocytes stimulated with the toll-like receptor agonists LPS and R848, in addition to prolonging monocyte survival. Both effects required the Fc receptor CD32a. The transcriptional response of monocytes cultured with anti-MPO IgG was directed towards the expression of extracellular matrix and extracellular proteins. These observations give insights into mechanisms of inflammation and fibrosis in ANCA vasculitis. We have produced a small list of candidate transcipts which are a clear basis for further study.

## Author contributions

MR designed the research study, analysed data, and wrote the manuscript. FFB and SB performed experiments and analysed data. PP analysed data**.**

## Declaration of competing interest

The authors have declared that no conflict of interest exists.

## Data Availability

Raw data will be shared following any reasonable request..
