# Supplementary material for: Antimyeloperoxidase antibodies modulate inflammatory responses and activate profibrotic pathways in human monocytes
Source: J Autoimmun. 2023 Sep;139:103060. doi: 10.1016/j.jaut.2023.103060 (PMC10828547; doi:10.1016/j.jaut.2023.103060)
Supplement: Multimedia component 1 [file mmc1.pdf]

Supplementary Data

Table S1
Anti-MPO and Anti-PR3 levels were measured on purified IgG preparations used in this study. All samples were diluted to 3.5mg/ml before analysis. Demographics for the patients are shown. Control samples were from healthy laboratory workers. BVAS; Birmingham vasculitis activity score.

Anti-MPO IU/mL	Anti-PR3 IU/mL	Age	Gender	Renal BVAS	Total BVAS
MPO-ANCA					
109	0.3	80	M	12	21
54	<0.2	51	M	12	21
81	<0.2	23	F	12	12
76	<0.2	76	M	12	12
>134	0.9	59	F	12	20
156	0.4	62	M	12	19
47	0.3	75	M	12	18
1.8	<0.02	73	M	12	18
PR3-ANCA					
<0.2	13	26	M	12	27
0.5	13	86	M	12	21
0.3	39	72	M	12	23
<0.2	23	52	F	12	27
0.5	35	73	F	12	21
0.4	>177	77	M	12	24
0.3	10	68	M	12	33
1	18	76	M	12	27
Controls					
<0.2	<0.2				
1.3	<0.2				
<0.2	<0.2				
0.3	0.4				
<0.2	<0.2				
<0.2	<0.2				
0.2	<0.2				
<0.2	<0.2				

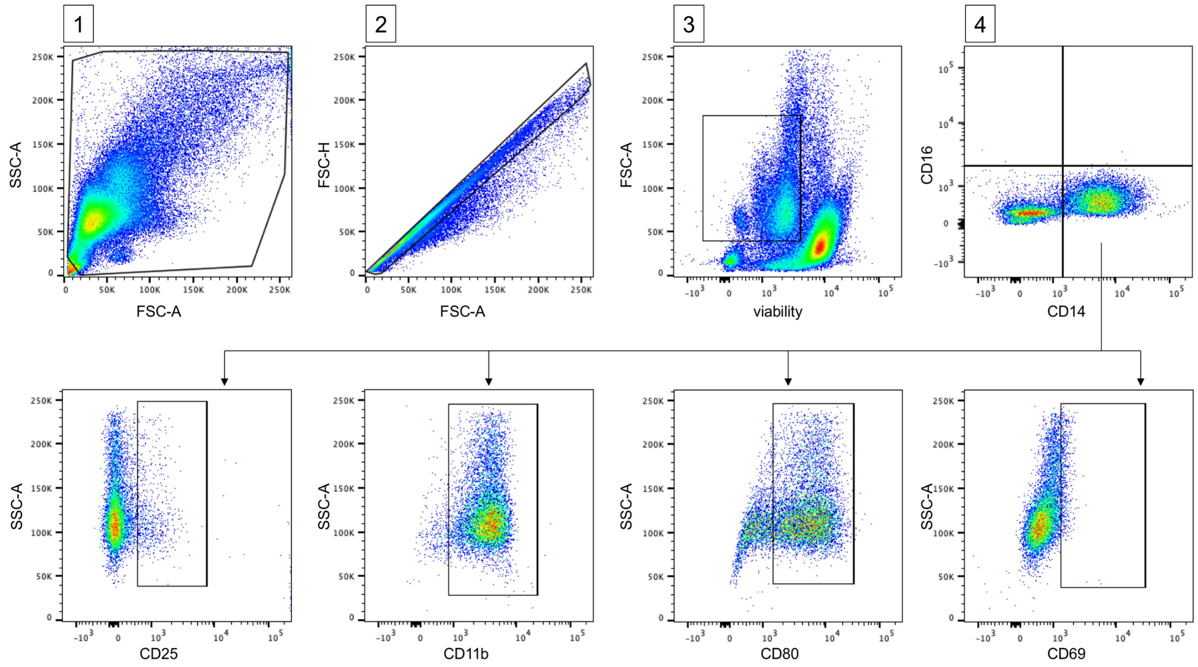

Figure S2. Gating strategy for the flow cytometric analysis of human peripheral blood monocytes stimulated for 18 hours with TLR agonists (LPS or R848) and IgG (anti-MPO, anti-PR3 or control). Data are presented in Figure 2.

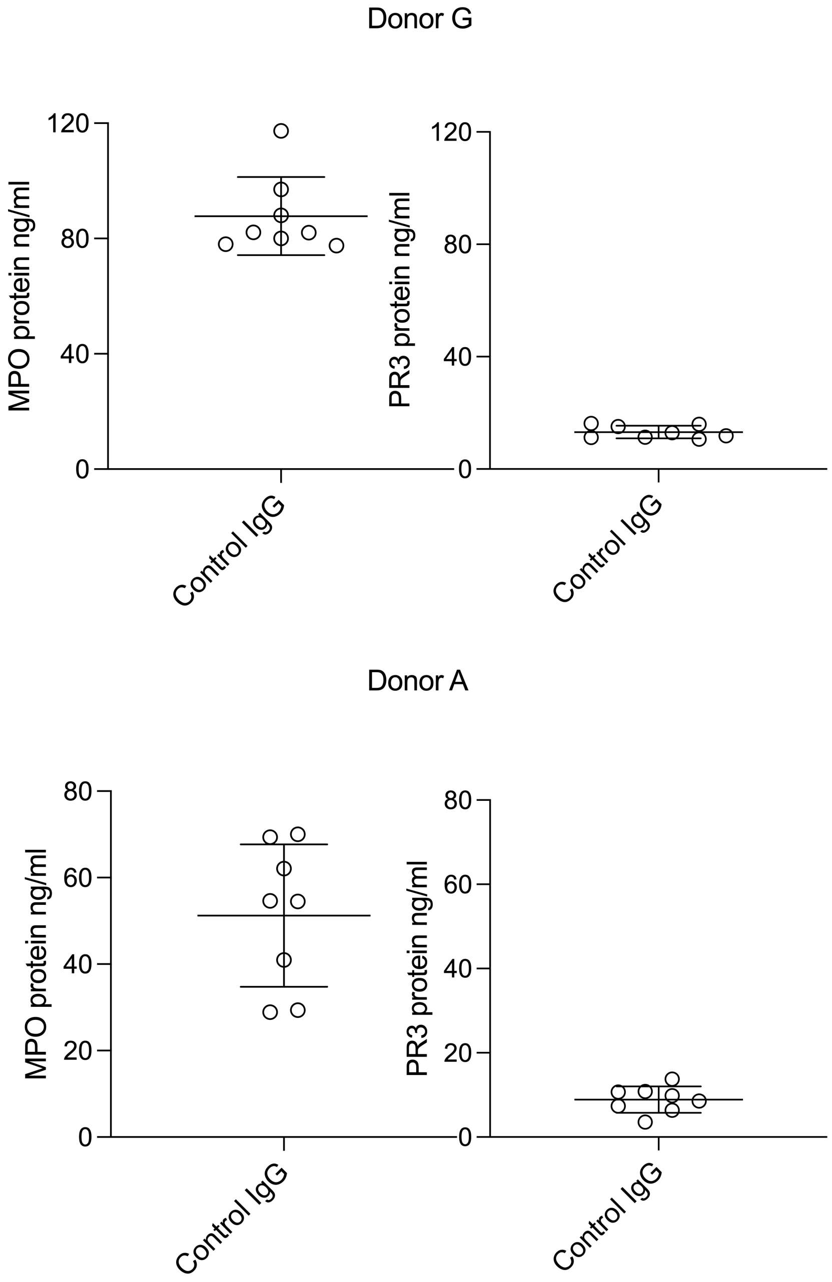

Figure S3. Human peripheral blood monocytes were cultured with control IgG (n=8 per group) for 6 days. MPO and PR3 protein levels were measured in the supernatants in two experiments with monocytes from donor G and donor A respectively. Symbols represent different IgG preparations and not technical replicates. Control IgG is from healthy donors. Error bars are mean (SD).

A

Donor A

	Without anti-CD32a				With anti-CD32a			
	Mean Log2 Counts				Mean Log2 Counts			
Transcript	Control	Anti-MPO	p value	q value	Control	Anti-MPO	p value	q value
**CXCL5**	5.972	7.701	0.000058	0.000091	5.977	6.884	0.002339	0.005731
**DUSP4**	7.911	9.567	0.000167	0.000145	7.941	8.29	0.135458	0.09051
**EGR2**	8.808	10.23	0.001359	0.000476	9.037	9.592	0.022672	0.023806
**EGR3**	8.279	10.27	0.000032	0.000091	8.342	9.052	0.046404	0.037897
**GEM**	7.974	9.986	0.000503	0.000226	7.995	8.486	0.064345	0.047294
HBEGF	4.077	7.468	0.048617	0.01178	1.388	5.886	0.018219	0.022319
**LINC00659**	6.715	8.828	0.000275	0.000145	6.457	7.418	0.008433	0.012396
**RGS1**	10.59	12.14	0.000276	0.000145	10.49	10.64	0.519281	0.293593
**RP11-667K14.3**	6.418	9.007	0.000218	0.000145	6.341	7.34	0.042012	0.037897
**SOWAHC**	7.321	8.313	0.002363	0.000677	7.414	7.774	0.005619	0.010325
SPRY2	2.746	8.516	0.011892	0.003122	0	6.356	<0.000001	0.000004
**THBD**	7.261	8.198	0.000902	0.000355	7.273	7.454	0.274736	0.168276
TRIB3	5.902	6.853	0.001642	0.000517	5.755	5.955	0.002106	0.005731

B Donor A

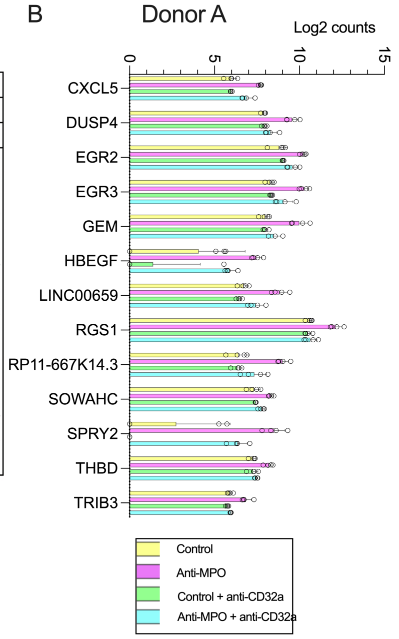

Figure S4. nCounter Nanostring analysis of RNA from human peripheral blood monocytes stimulated for 6 hours with R848 and anti-MPO or control IgG, in the presence or absence of anti-CD32a (n=4 per group). A custom code set including the 17 transcripts that were identified as differentially upregulated by anti-MPO IgG in donors D and F in the presence of both LPS and R848 (Figure 5B) was used. A. Mean Log2counts, p value and adjusted p values (q values) are shown for the transcripts where both p and q values were less than 0.05. Bold text indicates transcripts where the p value was at least two-fold higher with anti-CD32a. B. Graphical representation of the data. Error bars are mean (SD). P values were obtained using unpaired t tests with Benjamini, Krieger, and Yekutieli's correction for false discovery used to obtain q values.

A

Donor A

	Without anti-CD32a				With anti-CD32a			
	Mean Log2 Counts				Mean Log2 Counts			
Transcript	Control	Anti-MPO	p value	q value	Control	Anti-MPO	p value	q value
**CCND1**	0.8889	5.433	0.00930	0.018446	0	1.163	0.149688	0.176819
**CCND2**	1.022	5.884	0.01083	0.018446	5.886	4.793	0.429593	0.369059
**CD1B**	0	6.654	0.00048	0.008821	1.341	6.064	0.002016	0.009528
**CRABP2**	1.179	5.972	0.01784	0.024565	0	1.896	0.059729	0.103181
**CSF1***	1.435	9.641	0.00117	0.008821	0.6711	6.418	0.000192	0.003631
FLT1	0.9716	6.226	0.00806	0.018446	3.677	4.206	0.715583	0.563521
**GAL**	1.272	7.885	0.00913	0.018446	0	2.839	0.020566	0.048586
**GEM**	0.8288	4.884	0.01045	0.018446	0.6615	0	0.334282	0.300854
**GIPC3**	0.9019	5.108	0.01168	0.018446	0	0.6301	0.334282	0.300854
**IL1RN***	2.637	10.54	0.00455	0.018446	1.416	7.313	0.000943	0.005942
**IL36B**	0.9831	5.946	0.00926	0.018446	0	1.961	0.060052	0.103181
**INHBA***	1.038	6.1	0.01055	0.018446	0	3.308	0.004243	0.013768
**OCSTAMP**	1.045	6.504	0.00886	0.018446	0	0.5591	0.334282	0.300854
ROR1-AS1	0	4.015	0.004971	0.018446	1.383	0	0.14948	0.176819
**RP11-1008C21.1***	1.061	6.298	0.00982	0.018446	0	2.752	0.019556	0.048586
**RP11-20G13.2**	1.577	9.196	0.01025	0.018446	4.579	7.132	0.108428	0.157638
**RP11-20G13.3***	1.246	7.002	0.01192	0.018446	0	3.806	0.004371	0.013768
RPLP0P2	0.821	5.192	0.00929	0.018446	0	0		
**SCG5**	1.26	7.043	0.01218	0.018446	0	2.094	0.059676	0.103181
**SPOCD1**	0	3.295	0.01936	0.025499	0	0		
**TGM2***	1.17	8.162	0.00102	0.025499	0	4.759	0.000541	0.005115
**TNFSF15**	0.9296	7.364	0.00066	0.008821	0.8501	3.3	0.084172	0.132571
**USP2**	0.9186	5.015	0.01330	0.019195	0	0.6104	0.334282	0.300854

Donor B

	Without anti-CD32a				With anti-CD32a			
	Mean Log2 Counts				Mean Log2 Counts			
Transcript	Control	Anti-MPO	p value	q value	Control	Anti-MPO	p value	q value
**CCND1**	0	5.608	0.00002	0.00020	0	0.4923	0.334282	0.548605
**CCND2**	0	5.87	0.00003	0.00020	0	0.7359	0.334282	0.548605
**CD1B**	0	4.998	0.00091	0.00144	0.6107	1.632	0.421473	0.608125
**CRABP2**	1.242	6.249	0.00140	0.00158	0	0.5285	0.334282	0.548605
**CSF1**	7.142	10.43	0.00126	0.00155	3.646	4.283	0.706833	0.860637
**GAL**	0	6.179	0.00007	0.00028	0	0.7472	0.334282	0.548605
**GEM**	2.1	6.419	0.00879	0.00629	1.78	0	0.060017	0.303083
**GIPC3**	0	3.789	0.00058	0.00115	0	0		
GREM1	6.346	9.925	0.00411	0.00376	1.89	0.482	0.197841	0.548605
**IL1RN**	9.179	12.27	0.00017	0.00053	5.887	5.452	0.80951	0.860637
**IL36B**	2.233	7.171	0.00625	0.00468	3.275	0.562	0.028649	0.289353
IL36RN	1.238	4.721	0.02099	0.01377	1.247	0	0.148915	0.501348
**INHBA**	2.836	7.779	0.01239	0.00849	0.8061	0.5285	0.777573	0.860637
ITGB3	0	4.887	0.00109	0.00155	0	0		
**OCSTAMP**	0.5925	6.091	0.00411	0.00376	0	0.5325	0.334282	0.548605
**RP11-1008C21.1**	0	6.645	0.00004	0.00020	1.743	0.6887	0.353063	0.548605
**RP11-20G13.2**	7.075	9.523	0.00089	0.00144	2.83	6.103	0.080175	0.323908
**RP11-20G13.3**	0.6254	5.96	0.00043	0.00112	0	2.312	0.059771	0.303083
S100A16	0	3.343	0.00472	0.00384	0	0		
**SCG5**	4.996	10.2	0.00128	0.00155	0.7316	0.6148	0.904465	0.913509
**SPOCD1**	0	3.851	0.00487	0.00384	0	0		
**TGM2**	2.039	7.236	0.00355	0.00373	1.192	0.8112	0.740796	0.860637
**TNFSF15**	1.865	7.426	0.00429	0.00376	1.165	0.6173	0.585833	0.788922
**USP2**	0	4.021	0.00057	0.00115	0	0		

B

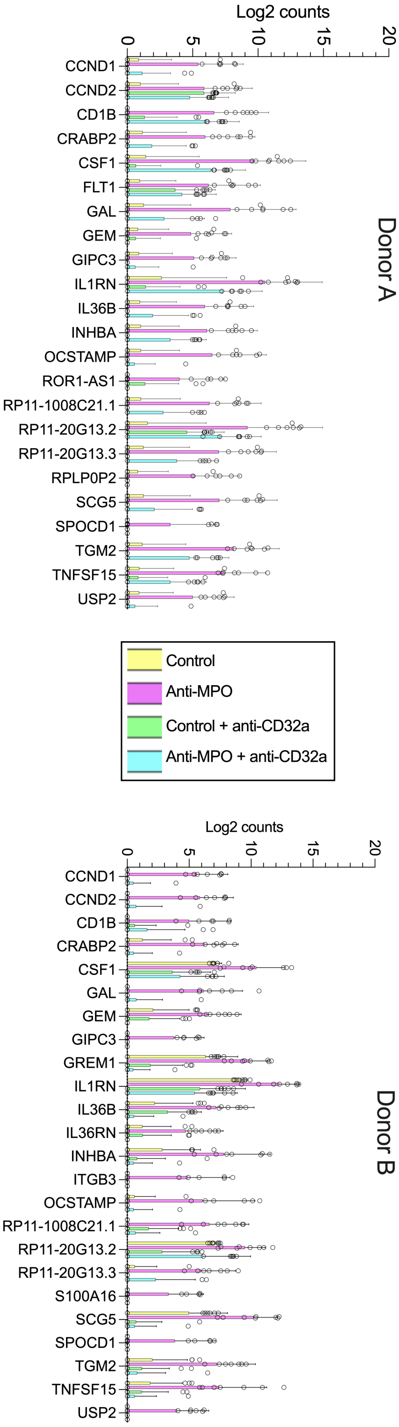

Figure S5. nCounter Nanostring analysis of RNA from human peripheral blood monocytes cultured for 24 hours with anti-MPO or control IgG, in the presence or absence of anti-CD32a (n=8 per group). A custom code set including the 45 transcripts that were identified as differentially expressed in donors B, G and A (Figure 6C) was used. For 3 of the 48 transcripts shown in 5C, probes were not available. A. Mean Log2 counts, p values and adjusted p values (q values) are shown for the transcripts where both p and q values were less than 0.05. Transcripts that were upregulated by anti-MPO IgG in both monocyte donors are shown in bold. An asterix denotes those where anti-CD32a did not increase the p value by at least two-fold. B. Graphical representation of the data. Error bars are mean (SD). P values were obtained using unpaired t tests with Benjamini, Krieger, and Yekutieli's correction for false discovery used to obtain q values.
